# Methyl Linderone Suppresses TPA-Stimulated IL-8 and MMP-9 Expression Via the ERK/STAT3 Pathway in MCF-7 Breast Cancer Cells

**DOI:** 10.4014/jmb.1911.11068

**Published:** 2019-12-30

**Authors:** Jae-Hwan Yoon, Thu-Huyen Pham, Jintak Lee, Jiyon Lee, Hyung-Won Ryu, Sei-Ryang Oh, Jae-Wook Oh, Do-Young Yoon

**Affiliations:** 1 Department of Bioscience and Biotechnology, Research Institute of Bioactive-Metabolome Network, Konkuk University, Seoul 05029, Republic of Korea; 2 Natural Medicine Research Center, Korea Research Institute of Bioscience and Biotechnology, Cheongju 8116, Republic of Korea; 3 Department of Stem Cell and Regenerative Biotechnology, Konkuk University, Seoul 05029, Republic of Korea

**Keywords:** Methyl linderone, metastasis, MCF-7 cells, IL-8, MMP-9

## Abstract

Methyl linderone (ML), a cyclo-pentenedione, was isolated from the fruit of *Lindera erythrocarpa* Makino (family Lauraceae). This plant has well-known anti-inflammatory effects; however, the anti-cancer effects of ML have not yet been reported. Thus, in the present study we investigated the effects of ML on the metastasis of human breast cancer cells. We used 12- O-tetradecanoyl phorbol-13-acetate (TPA)-stimulated MCF-7 cells as the cell model to study the effects of ML on invasion and migration. ML was found to reduce the invasion and migration rate of TPA-stimulated MCF-7 cells. Moreover, it inhibited two metastasis-related factors, matrix metalloproteinase-9 (MMP-9) and interleukin-8 (IL-8), at the mRNA and protein expression levels, in TPA-treated MCF-7 cells. The mechanism by which ML exerted these effects was through the inhibition of translocation of activator protein-1 (AP-1) and signal transducer and activator of transcription-3 (STAT3), mediated via phosphorylation of extracellular signal-regulated kinase (ERK). Taken together, our findings indicated that ML attenuated the TPA-stimulated invasion and migration of MCF-7 cells by suppressing the phosphorylation of ERK and its downstream factors, AP-1 and STAT3. Therefore, ML is a potential agent for the treatment of breast cancer metastasis.

## Introduction

Breast cancer is the second most prevalent cause of cancer mortality and the most common gynecological cancer worldwide [1, 2]. In 2018, there were approximately 2 million cases and 627,000 deaths and these numbers are predicted to increase yearly [3]. The breast cancer survival rate has risen recently because of improved diagnosis and treatment with a diverse range of anti-cancer agents. However, mortality rates remain high due to metastasis [4]. The metastatic process begins with cell invasion, which includes degradation of the extracellular matrix (ECM) and changes in cell adhesion, cell interactions, and inherent tissue structure [5]. The ECM is composed of collagen, elastin, and fibronectin, and it can be degraded by extracellular proteinases, such as matrix metalloproteinases (MMPs), which are metastasis-related factors in breast cancer [6, 7]. MMPs are a family of proteolytic enzymes consisting of zinc-dependent endopeptidases. They have been reported to be involved in rheumatoid arthritis and metastasis [8, 9]. The MMP family is divided into six subclasses: matrilysines (MMP-7 and -26), membrane-bound MMPs (MMP-14, -15, -16, -17, -24, and -25), stromalysines (MMP-3, -10, and -11), collagenases (MMP-1, -8, -13, and -18), gelatinases (MMP-2 and -9), and an unclassified group [10]. Among the various MMP groups, MMP-2 (gelatinase-A) and -9 (gelatinase-B) are the key enzymes associated with metastasis and invasion [11]. Therefore, suppressing MMP-9 expression is a potential approach to control malignant metastasis. Many stimulators, such as tumor necrosis factor-α, fibroblast growth factor-2, and 12-O-tetradecanoylphorbol-13-acetate (TPA) can induce MMP-9 overexpression [12, 13]. Among a variety of stimuli, TPA, as a tumor promoter, has been reported to induce MMP-9 expression by upregulating transcription factors, such as activator protein-1 (AP-1), signal transducer and activator of transcription-3 (STAT3), and nuclear factor kappa B (NF-κB) [14, 15]. TPA has also been shown to activate IL-8 expression and this model is widely used to assess carcinogenesis pathways [16].

Methyl linderone (ML) was initially isolated from the dried fruits of *Lindera erythrocarpa* Makino (family Lauraceae) [17]. This plant is used as a traditional medicine and is reported to have digestive, anti-bacterial, and anti-inflammatory effects [18]. However, the anti-migratory and anti-invasive effects of ML have not yet been fully examined. In this study, we evaluated the anti-metastatic effects and investigated the underlying mechanisms of ML in TPA-stimulated MCF-7 breast cancer cells.

## Materials and Methods

### Preparation of ML

*Lindera erythrocarpa* fruits were collected from Jeju Island, Korea, in October 2013. A voucher specimen (KRIB 0000372) was deposited in the Herbarium of the Korea Research Institute of Bioscience and Biotechnology (Korea). The target compounds were isolated from dried fruits of *L. erythorocarpa* as previously described [19]. Detailed information on the isolation procedure can be found in the Supplementary Material. Briefly, the extracts (770.0 g, yield 15.4%) were fractionated on a silica gel column (10 × 90 cm, JEO prep 60, 40-63 μm, 2.3 kg) and eluted using hexane-ethyl acetate mixtures (20:1→15:1→10:1→8:1→6:1→4:1→2:1→1:1) to give 10 pooled fractions. In fraction 8, ML was purified and identified by nuclear magnetic resonance (NMR); ultraviolet–visible spectrophotometry (UV); mass spectrometry (MS); and MS/MS, using high-resolution MS spectral data (HRMS).

### Reagents

The stock solution of ML (100 mM) in dimethyl sulfoxide was stored in the dark at 4°C and then diluted in Dulbecco's modified Eagle’s medium (DMEM; Hyclone, USA) immediately before use. The CellTiter 96 AQueous One Solution Cell Proliferation Assay reagent (3-[4,5-dimethylthiazol-2-yl]-5-[3-carboxymethoxyphenyl]-2-[4-sulfophenyl]-2H-tetrazolium [MTS]) was obtained from Promega (USA). NE-PER Nuclear and Cytoplasmic Extraction Reagents were supplied from Thermo Fisher Scientific (USA). RIPA buffer was obtained from DyneBio (Korea). The ERK inhibitor, PD98059, and antibodies specific for p-ERK, ERK, p-JNK, JNK, p-p38, NF-κB p65, c-Jun, c-Fos, STAT3, and PARP were purchased from Cell Signaling Technology (USA). The horseradish peroxidase-conjugated anti-rabbit IgG and anti-mouse IgG secondary antibodies were purchased from Millipore Sigma (USA). Antibodies specific for GAPDH, p38, and NF-κB were obtained from Santa Cruz Biotechnology (USA).

### Cell Lines

The human breast adenocarcinoma cell line, MCF-7, was obtained from the American Type Culture Collection (USA). Cells were cultured in DMEM containing 10% (v/v) heat-inactivated fetal bovine serum (Millipore Sigma) at 37°C in an atmosphere of 5% CO_2_.

### Cell Viability Assays

Cell viability was assessed using an MTS assay, according to the manufacturer’s instructions. Cells (1 × 10^3^ cells/well) were seeded in 96-well plates and then treated with various concentrations of ML and TPA (50 nM) for 24 h. Absorbance was measured at 492 nm using a microplate reader (Apollo LB 9110; Berthold Technologies, Germany).

### Cell Migration Assays

For migration assays, cells (6 × 10^6^ cells/ml) were seeded onto the upper chambers (Corning Inc., USA) in DMEM without serum and treated with ML for 2 h. To the lower chamber, 750 μl of TPA-treated serum-free DMEM (50 nM) was then added. After incubation for 24 h, non-migrated cells were removed from the chamber. Migrated cells were stained with a Diff-Quik Solution kit (Sysmex, Japan). The number of migrated cells was assessed using a microscope. Cells were then dissolved in 10% acetic acid (100 μl) and colorimetrically measured at 620 nm.

### Cell Invasion Assays

For invasion assay, cells (6 × 10^6^ cells/ml) were seeded onto upper chambers coated with 20 μl of Matrigel in DMEM without serum and then treated with ML for 2 h. To the lower chamber, 750 μl of TPA-treated serum-free DMEM (50 nM) was then added. After incubation for 24 h, non-invading cells were removed from the chamber. Invading cells were stained with a Diff-Quik Solution kit and were quantified using a microscope. The cells were then dissolved in 10% acetic acid (100 μl) and colorimetrically measured at 620 nm.

### Gelatin Zymography

 For gelatin zymography, cells (1 × 10^5^ cells/well) were seeded in 12-well plates and incubated until fully grown. After an overnight incubation, cells were pretreated with ML for 2 h and then treated with TPA (50 nM) for 24 h. Forty microliters of culture supernatant was loaded on 10% SDS-polyacrylamide gels containing 0.1% gelatin. Gels were stained with InstantBlue (Millipore Sigma) for 1 h in the dark. Areas of gelatinolytic degradation were observed on the dark blue background.

### Western Blotting Analysis 

Cells were treated with various ML concentrations for 2 h and then with TPA for 24 h. They were then harvested and lysed in RIPA buffer containing a protease inhibitor cocktail (Roche Diagnostics, Germany). The nuclear and cytoplasmic components were fractionated using NE-PER Nuclear and Cytoplasmic Extraction Reagents, according to the manufacturer’s instructions. The protein content of the extracts was quantified using the Bradford assay (BioRad, USA). Cell lysates were separated by 10-15% SDS polyacrylamide gel electrophoresis. The proteins were then transferred onto polyvinylidene difluoride membranes (Millipore Sigma), which were blocked in 5% powdered skim milk, in Tris-buffered saline containing 0.1% Tween-20, for 1 h at RT. Membranes were then incubated overnight at 4°C with specific primary antibodies. After washing, the membranes were incubated with secondary antibodies for 1 h at room temperature. The results were visualized using a chemiluminescence detection kit (Advanstar, USA).

### qRT-PCR

Cells were incubated for 24 h and then treated with various concentrations of ML for 2 h and with TPA (50 nM) for 24 h. Cells treated with ML were harvested and RNA was extracted using an easy-BLUE Total RNA Extraction Kit (Intron Biotechnology, Korea), according to the manufacturer’s instructions. cDNA was reverse transcribed from the RNA samples using M-MuLV reverse transcriptase (New England Biolabs, USA). qPCR was evaluated using the SensiFAST SYBR NO-ROX Kit (Bioline, UK) and a Rotor-Gene 6000 series thermal cycler (software v1.7; Qiagen, Germany). Each sample contained one of the following primer sets: *MMP*-9, 5’- AATCTCACC GACAGGCAGCT-3’ (forward), 5’- CCAAACTGGATGACGTGTC-3’ (reverse); *IL*-8, 5’-CTTGGCAGCCTTCCTGATTT-3’ (forward), 5’- CTCAGCCCTC TTCAAACT-3’ (reverse); or *GAPDH*, 5’- TGATGACATCAAGAA GGTGGT-3’ (forward), 5’- TCCTTGGAGGCC TGT AGGCC-3’ (reverse).

### Statistical Analysis

Data are presented as means ± SD (*n* = 3). One-way analysis of variance, followed by Tukey’s HSD test, were used to evaluate differences between groups. Values of *p* < 0.05 or < 0.01 were considered statistically significant.

## Results

### Effects of ML on MCF-7 Cell Viability

**Fig. 1:**
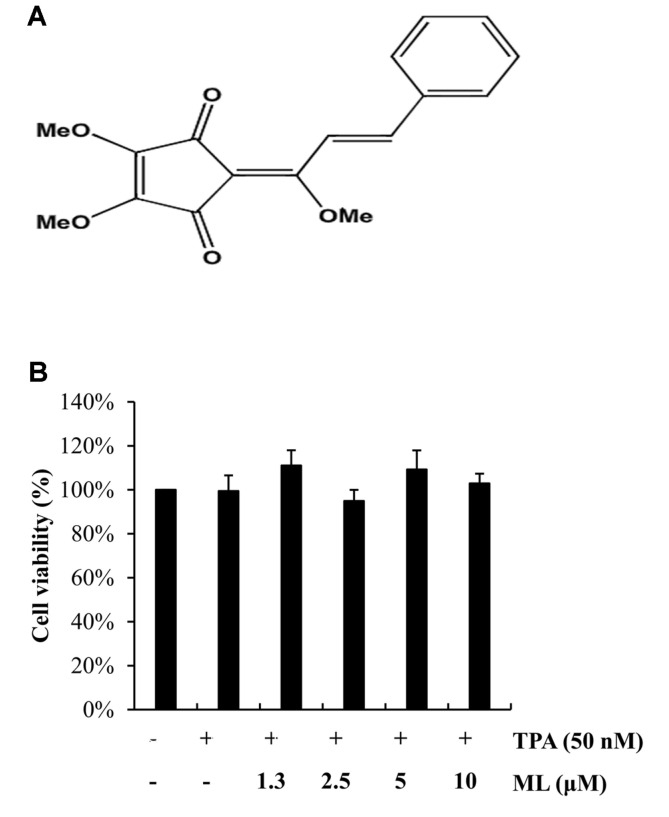
Chemical structure and cytotoxic effect of methyl linderone on TPA-stimulated MCF-7 cells. (**A**) Chemical structure of methyl linderone (ML). MW = 300.0997 (C_17_H_16_O_5_). (**B**) MCF-7 cells were pretreated with 1.3, 2.5, 5, or 10 μM ML for 2 h. After pretreatment, cells were treated with TPA (50 nM) in the presence or absence of ML. Cell viability was assessed using an MTS assay. Results represent the mean ± SD of three experiments.

The non-cytotoxicity of ML on MCF-7 cells was assessed using an MTS assay, in the presence or absence of TPA (50 nM) for 24 h. The results showed that ML did not affect the viability of MCF-7 cells up to a concentration of 10 μM (Fig. 1). Therefore, non-cytotoxic concentrations of ML up to 10 μM were used in subsequent experiments, to study its anti-invasive and anti-migratory effects.

### Effects of ML on the Invasion and Migration of TPA-Stimulated MCF-7 Cells

**Fig. 2:**
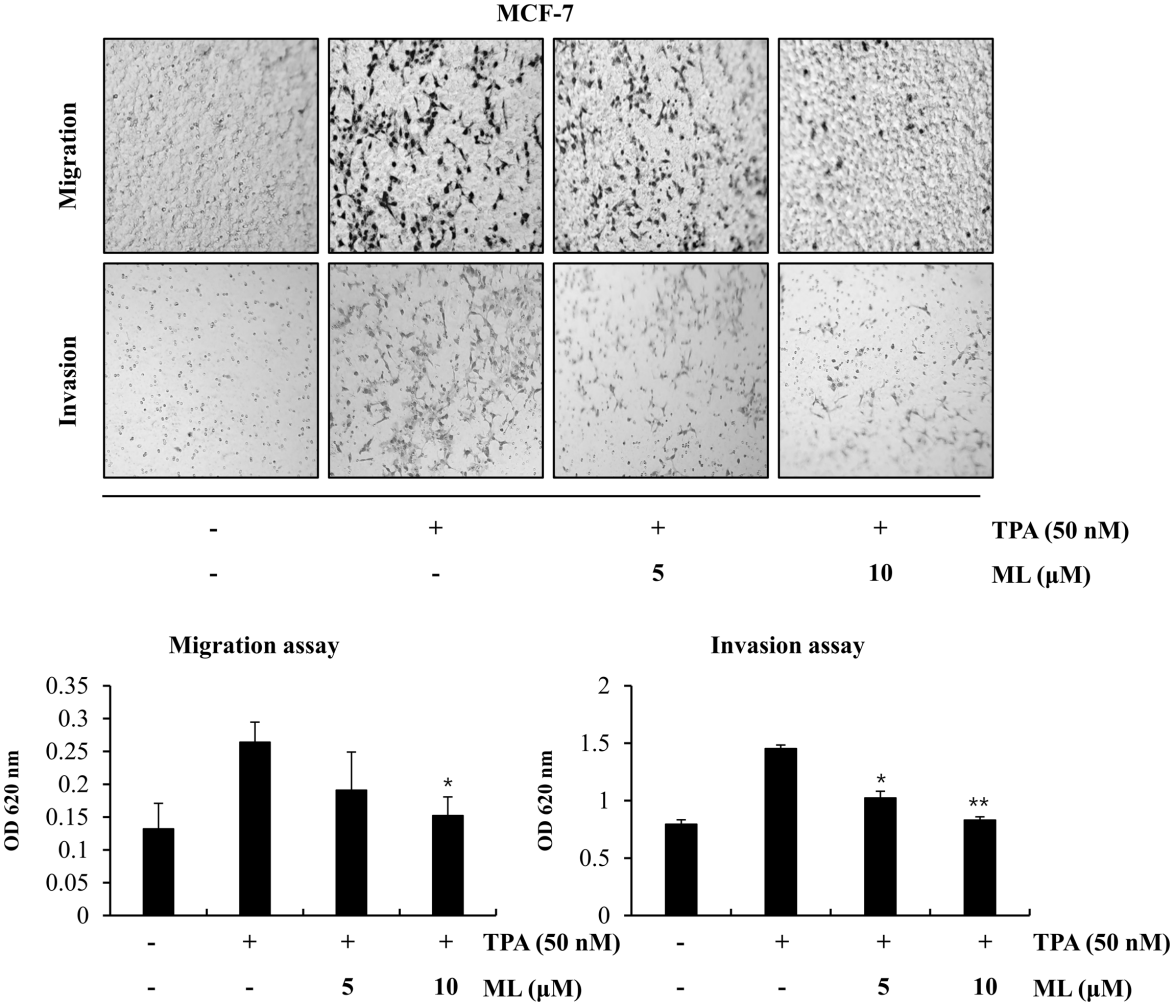
Effects of ML on breast cancer cell migration and invasion. For migration assays, MCF-7 cells, without Matrigel, were pretreated with ML at the indicated concentrations and then treated with TPA (50 nM). For invasion assays, cells, with Matrigel, were treated with ML and TPA (50 nM). Results represent the mean ± SD of three experiments (TPA alone vs. TPA plus ML, * *p* < 0.05, ** *p* < 0.01).

To evaluate whether ML had anti-metastatic properties, MCF-7 cells were pre-treated with ML for 2 h and then with TPA for 24 h. Invasion and migration rates were then assessed using Matrigel invasion and migration assays. As shown in Fig. 2, TPA-stimulated cell migration was inhibited by ML in a dose-dependent manner. Moreover, ML attenuated the invasion of TPA-stimulated MCF-7 cells (Fig. 2).

### Effect of ML on MMP-9 Expression in TPA-Stimulated MCF-7 Cells

**Fig. 3:**
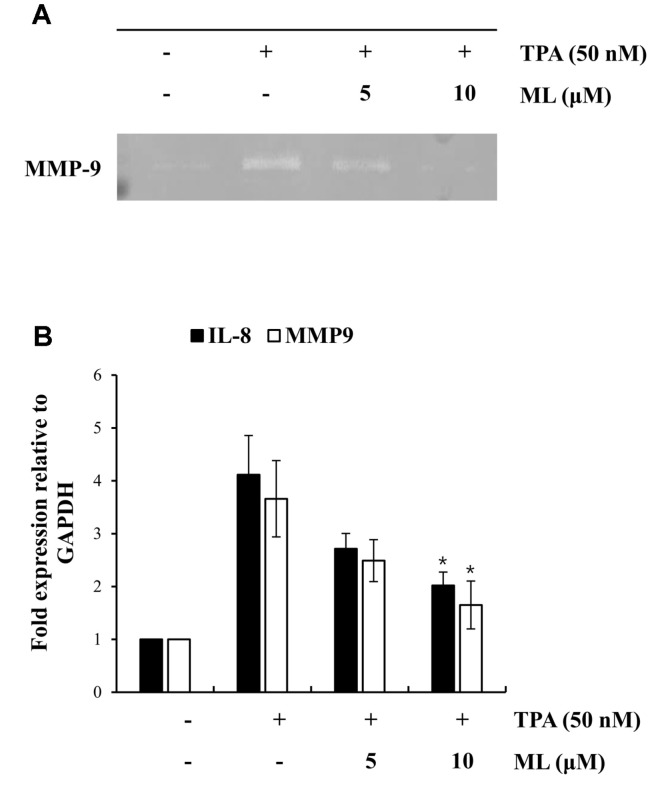
Effects of ML on MMP-9 activity and the mRNA expression of *IL*-8 and *MMP*-9. Cells were pretreated with ML (5 or 10 μM) for 2 h and then with TPA (50 nM) for 24 h. MMP-9 activity after TPA stimulation was assessed using gelatin zymography (**A**). mRNA expression of *IL*-8 and *MMP*-9 was assessed by RT-qPCR (**B**). Results represent the mean ± SD of three experiments (TPA alone vs. TPA plus ML, * *p* < 0.05).

To elucidate whether ML could inhibit TPA-stimulated MMP-9 and IL-8 expression in MCF-7 cells, their mRNA expression levels were assessed by qRT-PCR. *MMP*-9 and *IL*-8 mRNA levels decreased with ML treatment, in a dose-dependent manner (Fig. 3). Moreover, the zymography results indicated that the enzymatic activity of MMP-9, which was induced by TPA treatment, was suppressed by ML in MCF-7 cells. As shown in Fig. 3, these results demonstrated that ML inhibited the enzymatic activity of MMP-9 and *MMP*-9 and *IL*-8 mRNA expression levels under TPA-stimulated conditions in MCF-7 cells.

### Effect of ML on ERK/MAPK in TPA-Stimulated MCF-7 Cells

**Fig. 4:**
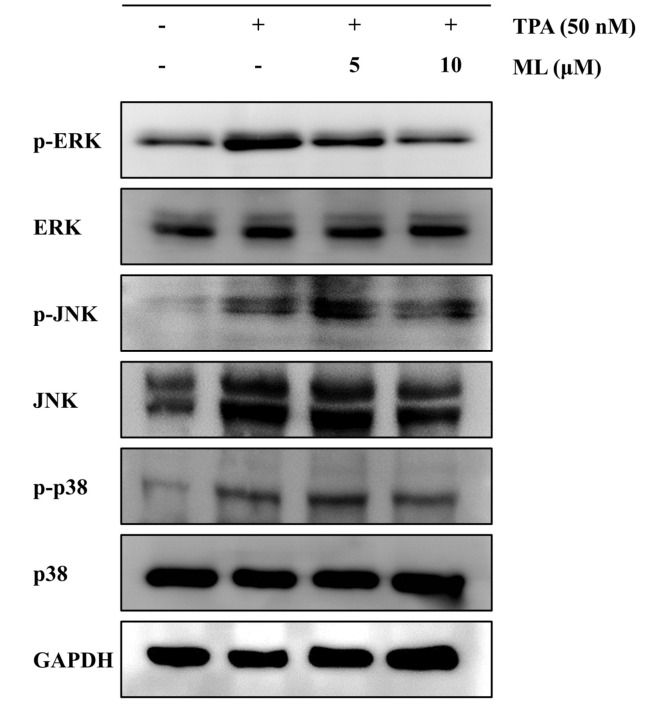
Effect of ML on MAP kinase in TPA-stimulated breast cancer cells. MCF cells were pretreated with ML (5 or 10 μM) and then with TPA (50 nM) for 24 h. Western blotting analyses of p-ERK, p-JNK, and pp38 were performed using whole-cell lysates.

Next, we assessed whether ML could affect MAPK signaling pathway factors, including ERK, JNK, and p38, which are involved in the regulation of the metastasis-related factors, IL-8 and MMP-9. TPA treatment was found to increase the phosphorylation of p-38, JNK, and ERK (Fig. 4). Only the phosphorylation of ERK was inhibited by ML in a dose-dependent manner, while the phosphorylation of JNK and p38 were not affected by ML (Fig. 4). Thus, TPA-stimulated phosphorylation of ERK was the target of ML in MCF-7 cells.

### Effects of ML on Nuclear Translocation of Transcription Factors

**Fig. 5:**
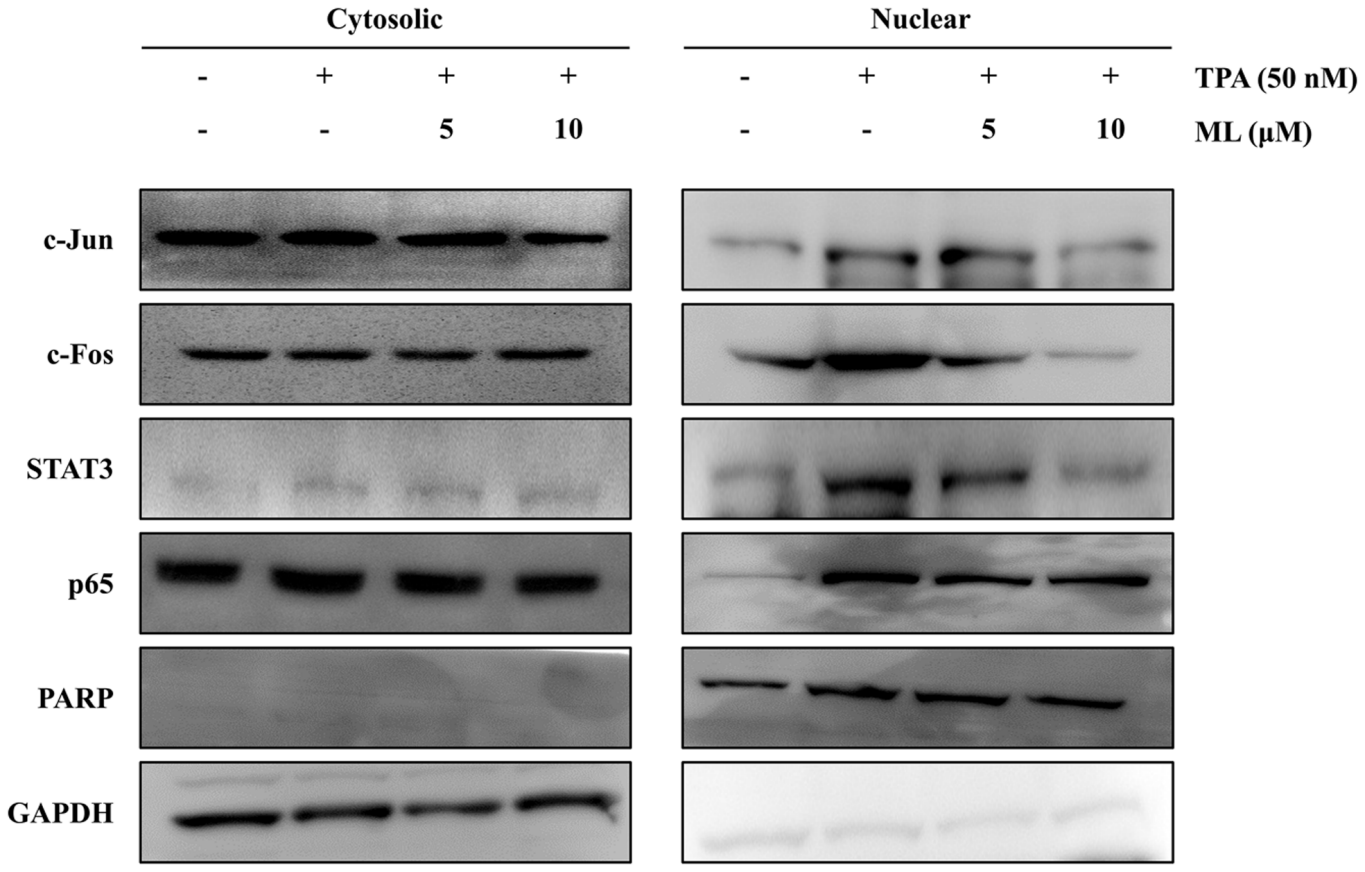
Effect of ML on transcription factors in TPA-stimulated MCF-7 cells. MCF-7 cells were pretreated with ML for 2 h and then treated with TPA. After 24 h of incubation, AP-1 and STAT3 levels were evaluated in nuclear and cytosolic fractions. The translocation of these factors was detected using specific antibodies. PARP and GAPDH were used as internal controls.

**Fig. 6:**
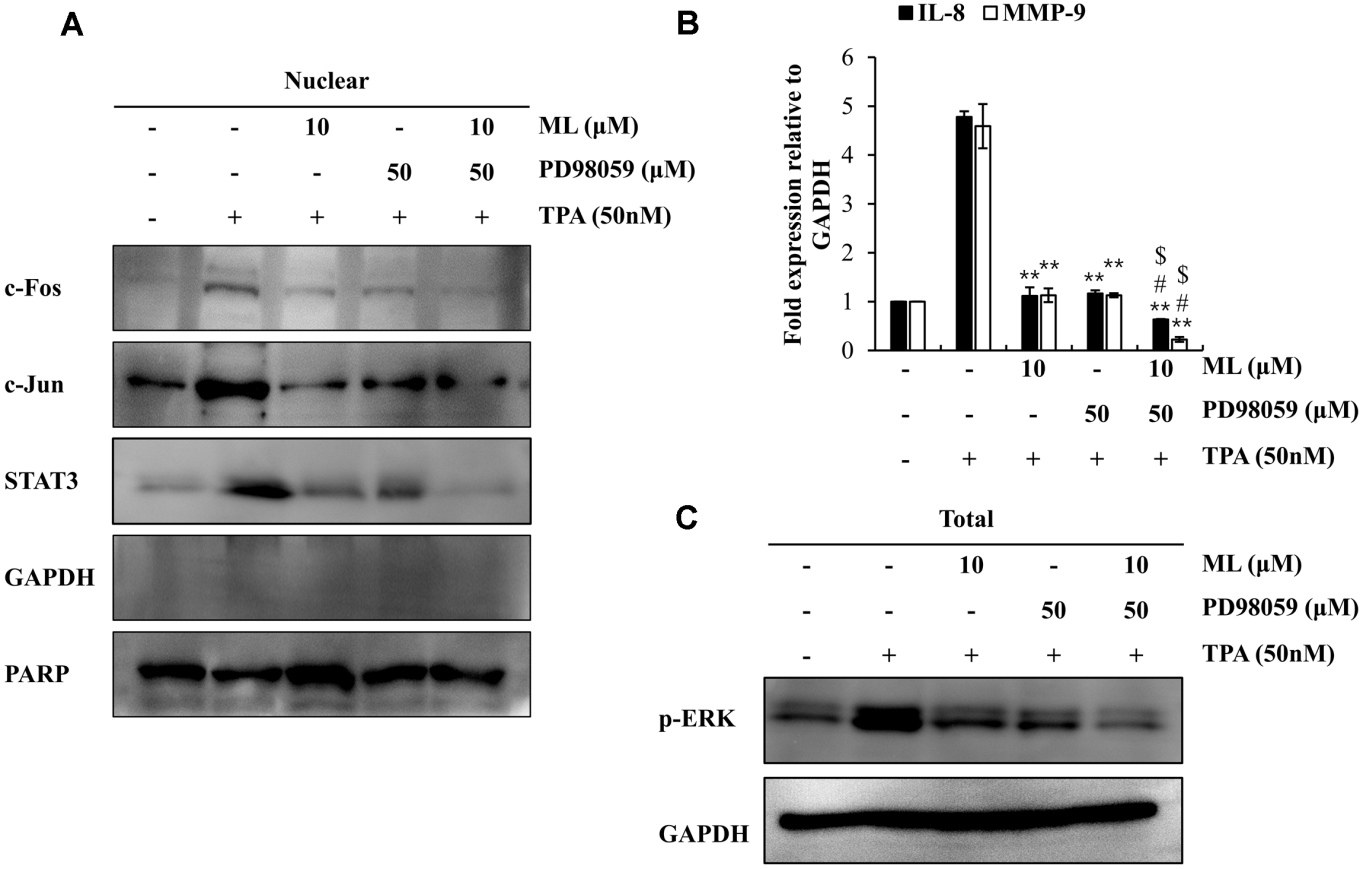
Effect of ML on the metastasis-related factor, ERK. MCF-7 cells were pretreated with ML in the presence or absence of the ERK inhibitor, PD98059 (50 μM), for 2 h. After incubation, cells were treated with TPA for 24 h. Nuclear transcription factors were assessed by nuclear fractionation and western blotting (**A**). *IL*-8 and *MMP*-9 mRNA expression levels were evaluated by qRT-PCR (**B**). p-ERK levels were evaluated by western blotting (**C**). Results represent the mean ± SD of three experiments (TPA alone vs. TPA plus ML or PD98059, **p < 0.01; TPA plus ML vs. TPA plus ML and PD98059, # *p* < 0.05; TPA plus PD98059 vs. TPA plus ML and PD98059, $ *p* < 0.05).

The expression of metastasis-related factors is affected by specific transcription factors. Therefore, to examine the effect of ML on the translocation of transcription factors, nuclear fractionation experiments were performed. The nuclear translocation of AP-1 (including c-Jun and c-Fos subunits) and STAT3 was found to be suppressed by ML in TPA-treated MCF7 cells (Fig. 5). The ERK inhibitor, PD98059, was used to confirm that ERK was the upstream regulator of STAT3 and AP-1 in the ML-triggered signaling pathway. ML was found to have similar inhibitory effects as PD98059, on the TPA-stimulated nuclear translocation of AP-1 and STAT3 (Fig. 6A). Furthermore, *IL*-8 and *MMP*-9 mRNA expression was inhibited in cells pre-treated with PD98059 or ML, prior to TPA-treatment (Fig. 6B). These data showed that ML attenuated *MMP*-9 and *IL*-8 expression by suppressing the ERK signaling pathway.

## Discussion

ML has been reported to regulate inflammation by suppressing nitric oxide synthesis, a mediator of acute inflammation, and to suppress the proliferation of H-*ras*-transformed rat-2 cells [17, 20]. However, the regulatory effects of ML on metastasis have not yet been fully demonstrated in human breast cancer. Therefore, we sought to evaluate in the current study whether ML could modulate the metastasis-related factors, IL-8 and MMP-9, to regulate the migration and invasion of breast cancer cells. Furthermore, the underlying mechanism by which ML may act in TPA-stimulated MCF-7 cells was investigated.

Metastasis is the leading cause of cancer-related mortality and it occurs frequently in breast cancer [21]. Tumor metastasis is complex and involves multiple processes, including ECM proteolytic degradation, cell invasion, and cell migration, resulting in cancer growth at the metastatic site [10]. MMP-9 is an important component of the basement membrane and has important functions in tissue recovery. However it can also support the metastatic features of cancer cells, helping them become motile and invasive [22, 23]. Previous studies have demonstrated the expression and activity of MMP-9 in a variety of human cancer cells, including breast cancer cells [23, 24]. Thus, targeting MMP-9 expression is a potential strategy to regulate tumor metastasis. In the present study, the TPA-stimulated migration and invasion of MCF-7 cells were inhibited by ML, via the suppression of MMP-9 expression and activity. IL-8, known as neutrophil chemotactic factor, also plays a key role in metastasis and is reported to be overexpressed in breast cancer cells after TPA treatment [16]. In the present study, ML was found to inhibit *IL*-8 expression in TPA-stimulated MCF-7 cells. These results confirmed that ML has potential anti-metastasis effects by reducing MMP-9 and IL-8 levels.

To assess the detailed mechanism underlying the anti-metastatic effects of ML, various transcription factors were investigated, including AP-1, STAT3, and NF-kB, which are known to be modulators of *IL*-8 and *MMP*-9 transcription [14, 25]. The TPA-stimulated nuclear translocation of AP-1 and STAT3 was attenuated by ML, suggesting that AP-1 and STAT3 are the main transcription factors involved in the signaling pathway regulated by ML. Moreover, MMP-9 and IL-8 expression has been reported to be modulated by the MAPK family (including ERK, JNK, and p38) via the downstream factors, AP-1 and STAT3 [14, 26]. Therefore, we demonstrated that ML suppressed *MMP*-9 and *IL*-8 expression by regulating the ERK signaling pathway.

In conclusion, ML significantly downregulated *MMP*-9 and *IL*-8 expression in TPA-stimulated MCF7 cells, by inhibiting the ERK/STAT3/AP-1-mediated signaling pathway. These results suggested that ML is a potential pharmacological agent for breast cancer treatment, to block cancer invasion and migration.

## Supplemental Materials



Supplementary data for this paper are available on-line only at http://jmb.or.kr.
