# Supplementary material for: Methyl Linderone Suppresses TPA-Stimulated IL-8 and MMP-9 Expression Via the ERK/STAT3 Pathway in MCF-7 Breast Cancer Cells
Source: J Microbiol Biotechnol. 2019 Dec 30;30(3):325–32. doi: 10.4014/jmb.1911.11068 (PMC9728293; doi:10.4014/jmb.1911.11068)
Supplement: Supplementary file 1 [file JMB-30-3-325-supple.pdf]

J. Microbiol. Biotechnol.
https://doi.org/10.4014/jmb.1911.11068

jmb

Materials and Methods

General experimental procedures

The compound was characterized using spectroscopic data, including ^{1}H, ^{13}C NMR, and HRMS and was compared with previously published data [1]. An ACQUITY UPLC™ system (Waters Corporation, Milford, MA, USA), equipped with a binary solvent delivery manager and a photodiode array (PDA) was used for ultra-performance liquid chromatography (UPLC) analysis. High-resolution mass spectrometry (HRMS) analysis was performed using a UPLC quadrupole time-of-flight mass spectrometer (UPLC-QTOF-MS) equipped with an electrospray ionization (ESI) interface (Waters Q-TOF PremierTM, Waters Corporation). Nuclear magnetic resonance (NMR) analysis was carried out using a Fourier Transform (FT)-NMR spectrometer (JEOL ECZ500R; JEOL Ltd., Tokyo, Japan) for 1D spectra (^{1}H NMR and ^{13}C NMR).

Plant material and preparation of *Lindera erythrocarpa* fruit

The fruit of *L. erythorocarpa* was resamping from Jeju Island, Southern Korea in October 2013 (by Dr. Jin-Hyub Paik). The collected raw materials were deposited in the Herbarium of the Korea Research Institute of Bioscience & Biotechnology (KRIBB, KRIB 0000372). The target compounds were isolated from dried fruits of *L. erythorocarpa*, as previously described [1]. Briefly, the extracts (770.0 g, yield 15.4%) were fractionated on a silica gel column (10 × 90 cm, JEO prep 60, 40-63 μm, 2.3 kg) and eluted using hexane-ethyl acetate mixtures (20:1→15:1→10:1→8:1→6:1→4:1→2:1→1:1) to give 10 pooled fractions. Fraction. 6 was subjected to high-performance liquid chromatography (HPLC) using a reversed-phase

column (YMC-Pack ODS-AQ-HG, 10 mm) and was eluted with a 70% MeOH isocratic system (flow: 100 mL/min, 55.0 min) by seven repeated sample injections (500 mg/8 mL methanol dilutions) to isolate methyl linderone (2.5 g).

Methyl linderone

The characteristics of methyl linderone were as follows: pale-yellow crystals; UV (MeOH) λ_{max} nm 240, 352; ^{1}H NMR (400 MHz, $CDCl_3$) δ 7.92 (1H, d, J = 16.0 Hz, H-α), 7.59 (2H, dd, J = 7.8, 2.1 Hz, H-2, 6), 7.50 (1H, d, J = 16.0 Hz, H-β), 7.36 (3H, m, H-3, 4, 5), 4.17 (6H, 2',3'-OMe), 4.08 (β'-OMe); ^{13}C NMR (100 MHz, $CDCl_3$) 60.0 (2',3'-OMe), 64.3 (β'-OMe), 109.4 (C-5'), 121.2 (C-α), 128.3 (C-2, 6), 128.9 (C-3, 5), 130.0 (C-β), 135.6 (C-1), 141.2 (C-4), 147.8 (C-3'), 149.0 (C-2'), 165.4 (C-β'), 184.7 (C-1'), 187.2 (C-4'), HRESIMS *m/z* $[M+H]^+$ 301.1064, (calculated for $C_{17}H_{17}O_5$, 301.1076).

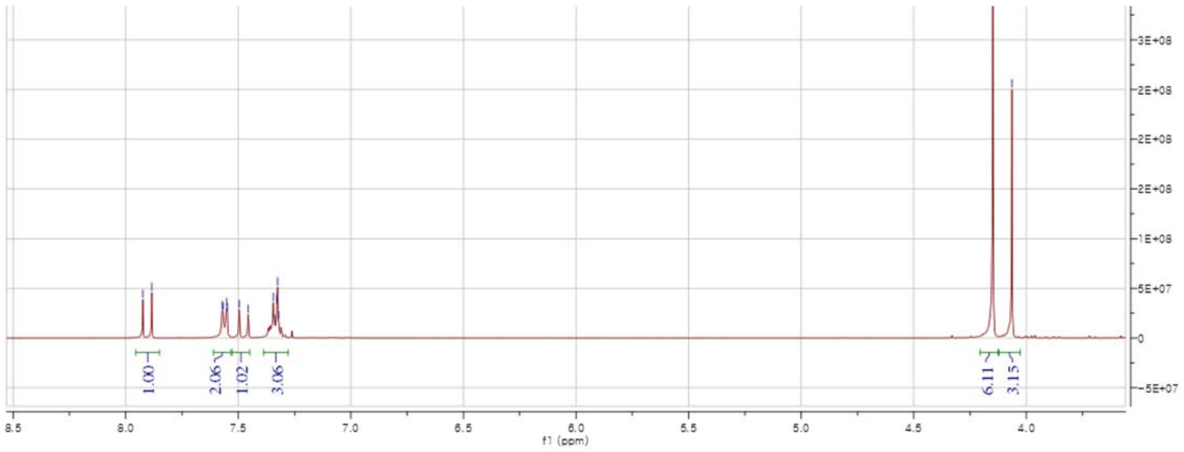

Figure S1. ^{1}H-NMR (400 MHz, $CDCl_3$) spectrum of methyl linderone

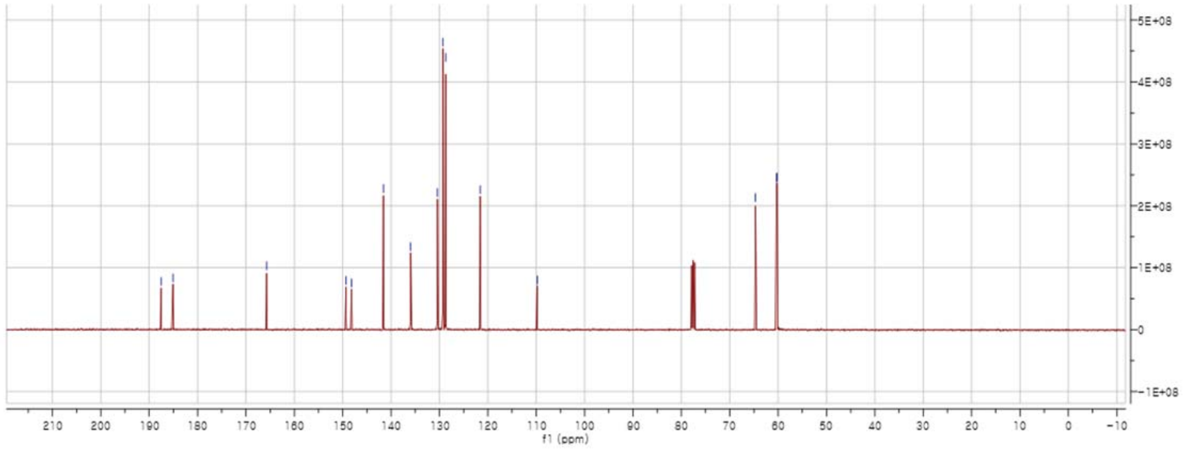

Figure S2. ^{13}C-NMR (100 MHz, $CDCl_3$) spectrum of methyl linderone

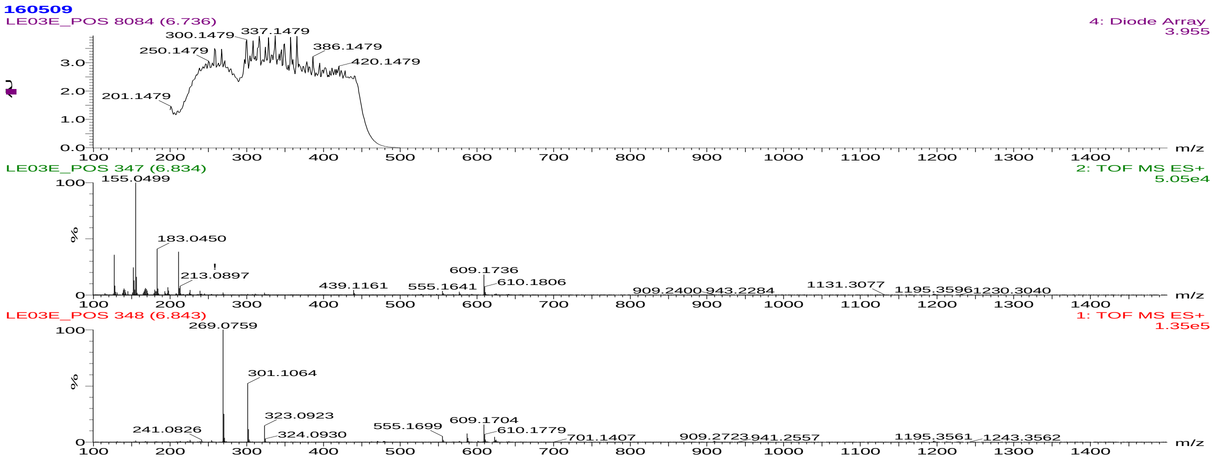

Mass	Calc. Mass	mDa	PPM	DBE	Formula	i-FIT	i-FIT Norm	Fit Conf %	C	H	O
301.1064	301.1076	-1.2	-4.0	9.5	C17 H17 O5	350.0	n/a	n/a	17	17	5

Figure S3. UV, MS^2, MS, and HR-ESI-MS data for methyl linderone

① Instrument

구 분	Instrument
HPLC	[Thermo] Dionex Ultimate 3000

② Solvents

구 분	Solvent
Solvent A	D.W
Solvent B	MeOH

③ Method – Gradient

Time(min)	Flow (mL/min)	%A	%B
(Initial)	1.000	50	50
5.00	1.000	50	50
25.00	1.000	0	100
30.00	1.000	0	100
31.00	1.000	50	50
35.00	1.000	50	50

④ Absorbance
UV : 280 nm, 254 nm, 230 nm, 360 nm

⑤ Inj. Volume
5 mg/ml, 5 μℓ

(6) Column
INNO Column

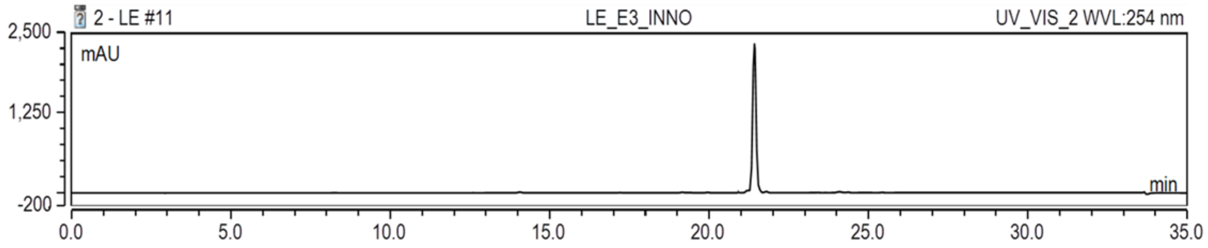

Figure S4. HPLC-DAD spectrum of isolated methyl linderone

Acknowledgements

This work was supported by the KRIBB Research Initiative Program funded by the Ministry of Science and ICT (MIST) of the Republic of Korea. We thank the Korea Basic Science Institute, Ochang, Korea, for providing the NMR data.

Reference

.

1. Lee SH, Oh HW, Fang Y, An SB, Park DS, Song HH, *et al.* 2015. Identification of plant compounds that disrupt the insect juvenile hormone receptor complex. *Proc Natl Acad Sci U S A*. **112:** 1733-1738.
